# Can the behavior of blood pressure after elevation of the positive end-expiratory pressure help to determine the fluid responsiveness status in patients with septic shock?

**DOI:** 10.5935/0103-507X.20200065

**Published:** 2020

**Authors:** Samia Cherem, Veviani Fernandes, Karine Duarte Zambonato, Glauco Adrieno Westphal

**Affiliations:** 1 Hospital Municipal São José - Joinville (SC), Brazil.

**Keywords:** Fluid responsiveness, Positive end-expiratory pressure, Arterial pressure/physiology, Pulse pressure respiratory variation, Shock, septic, Intensive care units, Fluido-responsividade, Respiração com pressão positiva, Pressão arterial/fisiologia, Variação respiratória da pressão de pulso, Choque séptico, Unidades de terapia intensiva

## Abstract

**Objective:**

To evaluate whether the decrease in blood pressure caused by the increase in the positive end-expiratory pressure corresponds to the pulse pressure variation as an indicator of fluid responsiveness.

**Methods:**

This exploratory study prospectively included 24 patients with septic shock who were mechanically ventilated and subjected to three stages of elevation of the positive end-expiratory pressure: from 5 to 10cmH_2_O (positive end-expiratory pressure level 1), from 10 to 15cmH_2_O (positive end-expiratory pressure level 2), and from 15 to 20cmH_2_O (positive end-expiratory pressure level 3). Changes in systolic blood pressure, mean arterial pressure, and pulse pressure variation were evaluated during the three maneuvers. The patients were classified as responsive (pulse pressure variation ≥ 12%) or unresponsive to volume replacement (pulse pressure variation < 12%).

**Results:**

The best performance at identifying patients with pulse pressure variation ≥ 12% was observed at the positive end-expiratory pressure level 2: -9% systolic blood pressure variation (area under the curve 0.73; 95%CI: 0.49 - 0.79; p = 0.04), with a sensitivity of 63% and specificity of 80%. Concordance was low between the variable with the best performance (variation in systolic blood pressure) and pulse pressure variation ≥ 12% (*kappa* = 0.42; 95%CI: 0.19 - 0.56). The systolic blood pressure was < 90mmHg at positive end-expiratory pressure level 2 in 29.2% of cases and at positive end-expiratory pressure level 3 in 41.63% of cases.

**Conclusion:**

Variations in blood pressure in response to the increase in positive end-expiratory pressure do not reliably reflect the behavior of the pulse pressure as a measure to identify the fluid responsiveness status.

## Abstract (pt)

**Objetivo:**

Avaliar se a diminuição da pressão arterial provocada pela elevação da pressão parcial positiva final corresponde à variação da pressão de pulso como indicador de fluido-responsividade.

**Métodos:**

Estudo de caráter exploratório que incluiu prospectivamente 24 pacientes com choque séptico ventilados mecanicamente e submetidos a três etapas de elevação da pressão parcial positiva final: de 5 para 10cmH_2_O (nível da pressão parcial positiva final 1), de 10 para 15cmH_2_O (nível da pressão parcial positiva final 2) e de 15 para 20cmH_2_O (nível da pressão parcial positiva final 3). Alterações da pressão arterial sistólica, da pressão arterial média e da variação da pressão de pulso foram avaliadas durante as três manobras. Os pacientes foram classificados como responsivos (variação da pressão de pulso ≥ 12%) e não responsivos a volume (variação da pressão de pulso < 12%).

**Resultados:**

O melhor desempenho para identificar pacientes com variação da pressão de pulso ≥ 12% foi observado no nível da pressão parcial positiva final 2: variação de pressão arterial sistólica de -9% (área sob a curva de 0,73; IC95%: 0,49 - 0,79; p = 0,04), com sensibilidade de 63% e especificidade de 80%. A concordância foi baixa entre a variável de melhor desempenho (variação de pressão arterial sistólica) e a variação da pressão de pulso ≥ 12% (*kappa* = 0,42; IC95%: 0,19 - 0,56). A pressão arterial sistólica foi < 90mmHg no nível da pressão parcial positiva final 2 em 29,2% dos casos e em 41,6,3% no nível da pressão parcial positiva final 3.

**Conclusão:**

Variações da pressão arterial em resposta à elevação da pressão parcial positiva final não refletem de modo confiável o comportamento da variação da pressão de pulso para identificar o *status* da fluido-responsividade.

## INTRODUCTION

Determining the patient’s capacity to respond to volume expansion is essential during the management of hemodynamically unstable patients in the intensive care unit (ICU)^(1,2)^ because it allows us to identify those who may benefit from volume infusion, as well as avoiding fluid overload in individuals already replete from a volume point of view.^(1,3)^ However, in clinical practice, the assessment of fluid responsiveness may be difficult.^(4)^

Different methods of dynamic assessment of volume responsiveness have been proposed in recent decades, with an emphasis on the pulse pressure respiratory variation (PPV) and systolic volume variation (SVV), both of which are parameters with high accuracy.^(1,3-5)^ The accuracy of these methods is impaired when there are cardiac arrhythmias, spontaneous ventilatory incursions, elevated positive end-expiratory pressure (PEEP), auto-PEEP, tidal volume < 8mL/kg or > 10mL/kg, abdominal hypertension, or severe pulmonary hypertension.^(1.5)^ In addition, the possible unavailability of equipment to monitor these parameters should also be considered.^(4.5)^

In 1999, Michard et al. observed that PPV could be used to monitor the hemodynamic effects of the application of PEEP, such as decreased cardiac output (CO) and blood pressure.^(6)^ More recently, it was observed that the drop in mean arterial pressure (MAP) caused by the increase in PEEP allows the identification of responsive patients.^(4)^ However, the application of high PEEP levels may result in severe hypotension or hypoxemia, especially in more hypovolemic patients.^(4.7)^ Considering that the use of lower levels of PEEP is safer, it was hypothesized that the generation of a gradient of 10cmH_2_O aimed at lower levels of final PEEP can help in the evaluation of fluid responsiveness.

The objective of this study was to evaluate whether the decrease in blood pressure caused by increased PEEP corresponds to PPV as an indicator of fluid responsiveness.

## METHODS

This is a prospective exploratory study conducted in the adult ICUs of the *Hospital Municipal São José* and the *Centro Hospitalar Unimed* in Joinville (SC), Brazil, from January to October 2018.

Patients older than 18 years diagnosed with septic shock, admitted to the ICU for up to 24 hours, under mechanical ventilation, and under the effect of analgesic and/or pharmacological muscle paralysis were considered eligible for the study and were included immediately after initial volume expansion.

The exclusion criteria were confirmed or suspected intracranial hypertension, heart disease (arrhythmias, valve diseases, or ventricular dysfunctions), pulmonary arterial hypertension, auto-PEEP or bronchospasm, abdominal compartment syndrome, and absence of invasive blood pressure monitoring.

The diagnosis of septic shock was made based on the coexistence of an infectious focus and the need for infusion of vasoactive amine.^(8)^

Patients were ventilated with a ventilator (CARESCAPE R860, GE HealthCare, Milwaukee, WI, USA) in controlled volume mode, with tidal volume (Vt) of 8mL/kg, 1:3 inspiration/expiration ratio, an inspired oxygen fraction (FiO_2_) that would maintain arterial oxygen saturation (SaO_2_) ≥ 92% and PEEP of 5cmH_2_O. The analgesic level and synchronization with mechanical ventilation were evaluated. If necessary, additional doses of analgesics, sedatives, or neuromuscular blockers were administered.

All patients were monitored with multiparameter monitors (MX500, Philips Medizim Systeme, Boeblingem, Germany), including continuous electrocardiography, pulse oximetry, central venous pressure (CVP), and invasive blood pressure measurement. The pressure transducers were zeroed at the mid-axillary line. The infusion rate of vasoactive amines was kept constant throughout the intervention period. The monitored variables were divided into ventilatory (Vt, PEEP, peak pressure - Pp, plateau pressure - Ppl, and SaO_2_) and hemodynamic variables (heart rate - HR, systolic blood pressure - SBP, MAP, CVP, and continuous automated PPV, which was calculated manually with the formula: $\mathrm{PPV}\left(\%\right)=100\times \left({\mathrm{Pp}}_{\max}-{\mathrm{Pp}}_{\min}\right)/\left.\left[\left({\mathrm{Pp}}_{\max}+{\mathrm{Pp}}_{\min}\right)/2\right]\right).$

### Definition of fluid responsiveness status

The CO was not evaluated, and the fluid-responsiveness status was inferred from the continuous PPV measurement. Based on the findings of a recent meta-analysis, a cut-off point of 12% was adopted to infer volume responsiveness.^(9)^ Thus, patients were classified as potentially responsive (PPV ≥ 12%) or potentially unresponsive to volume (PPV < 12%).

### Intervention

After adjusting the ventilatory parameters, performing initial volume expansion with 30mL/kg of crystalloids, complementing muscle sedation and/or paralysis if necessary, and estimating the fluid responsiveness status from the PPV, the patients were subjected to three distinct stages of airway pressurization by increasing the PEEP to three different levels: PEEP_1_ (level 1), corresponding to a PEEP increase from 5 to 10cmH_2_O for 60 seconds; PEEP_2_ (level 2), which was an increase in PEEP from 10 to 15cmH_2_O for 60 seconds; and PEEP_3_ (level 3), which was the increase in PEEP from 15 to 20cmH_2_O, also for 60 seconds.

The variation in blood pressure was calculated for each of the three PEEP levels, and the percentage variations in MAP (ΔMAP, %) and SBP (ΔSBP, %) were determined after PEEP application according to equations 1 and 2:

(Equation 1) $$\begin{array}{c} \left({\mathrm{PEEP}}_{\mathrm{highest}}\mathrm{MAP}-{\mathrm{PEEP}}_{\mathrm{lowest}}\mathrm{PAM}\right)/\\ \left[\left({\mathrm{PEEP}}_{\mathrm{highest}}\;\mathrm{MAP}+{\mathrm{PEEP}}_{\mathrm{lowest}}\mathrm{PAM}\right)/2\right]\times 100 \end{array}$$

(Equation 2) $$\begin{array}{c} \left({\mathrm{PEEP}}_{\mathrm{highest}}\mathrm{SBP}-{\mathrm{PEEP}}_{\mathrm{lowest}}\mathrm{SBP}\right)/\\ \left[\left({\mathrm{PEEP}}_{\mathrm{highest}}\mathrm{SBP}+{\mathrm{PEEP}}_{\mathrm{lowest}}\mathrm{SBP}\right)/2\right]\times 100 \end{array}$$

The maneuvers were interrupted, and their values were recorded whenever any of the following situations were detected for more than 30 seconds: HR < 60bpm or > 150bpm, MAP < 65mmHg or SBP < 90mmHg, SaO_2_ < 88%, and Ppl > 35cmH_2_O.

### Volume test

After recording the monitored hemodynamic variables (HR, MAP, SBP, CVP, PPV, ΔMAP, and ΔSBP), all patients with PPV ≥ 12% and clinical and/or laboratory signs of hypoperfusion received 500mL of crystalloid over 15 minutes. Five minutes after the end of the infusion, the PEEP elevation maneuvers were repeated at three levels, as was the recording of hemodynamic variables.

### Clinical and demographic information

The following variables were collected and recorded for analysis: sex, age, Simplified Acute Physiology Score (SAPS) version 3, type of shock, infectious site (eight pulmonary, 11 abdominal, three urinary, and two cutaneous), hemodynamic variables (HR, MAP_i_, ΔMAP, PPV, and CVP) and ventilatory variables (V_t_, PEEP, Pp, Ppl, ΔCO_2_, central venous oxygen saturation - ScvO_2_, and SaO_2_).

### Statistical analysis

The statistical software MedCalc version 16.4.3 (MedCalc Software bvba, Ostend, Belgium) was used for statistical analysis. Continuous variables are expressed as median and interquartile range (IQR) and were compared with Student’s *t*-test when the sample distribution was normal, as shown by the Kolmogorov-Smirnov test. The Mann-Whitney test was used for nonnormally distributed variables. Categorical variables are expressed as raw number and percentage and were compared with Pearson’s chi-squared test. A value of p < 0.05 was considered statistically significant. The correlation and agreement of PPV with ΔSBP and ΔMAP in the fluid-responsiveness evaluation were determined with the Pearson correlation coefficient and Cohen’s kappa index, respectively.

We constructed receiver operating characteristic (ROC) curves for ΔMAP and ΔSBP at each of the PEEP elevation levels to identify the best cutoff values that corresponded to 12% PPV, as well as the corresponding sensitivity and specificity. Areas under the ROC curve (AUC) of 0.70 to 0.79 indicate moderate discriminatory capacity, and AUC ≥ 0.80 indicates excellent discrimination.^(10)^

The study was approved by the Research Ethics Committee of the *Hospital Municipal São José* (CAAE: 88510818.1.0000.5362), and the Informed Consent Form was obtained from each patient or guardian family member.

## RESULTS

Twenty-four patients with septic shock were analyzed, whose clinical and demographic information is shown in table 1. Of these, 13 had PPV ≥ 12% and 11 had PPV < 12% at the time of inclusion in the study.

**Table 1:** Characteristics of the patients

Characteristics	All (n = 24)	ΔPp ≥ 12% (n = 13)	ΔPp < 12% (n = 11)	p value
Male sex	13 (54.1)	9 (69.2)	4 (36.4)	0.10
Age (years)	56 (44 - 55)	57 (44 - 67)	53 (49 - 58)	0.39
SAPS 3	83 (71 - 88)	83 (69 - 85)	81 (71 - 89)	0.34
Infectious site				
Pulmonary	8 (33.3)	3 (23.1)	5 (45.5)	0.24
Abdominal	11 (45.8)	8 (69.2)	2 (18.2)	0.03
Urinary	3 (12.5)	1 (7.7)	2 (18.2)	0.43
Skin	2 (8.3)	1 (7.7	2 (18.2)	0.43
SaO_2_ (%)	97 (96 - 97)	96 (94 - 97)	97 (96 - 98)	0.86
Peak pressure (cmH_2_O)	22 (19 - 24)	22 (20 - 26)	21 (18 - 24)	0.42
Plateau pressure (cmH_2_O)	15 (13 - 18)	16 (14 - 19)	15 (13 - 17)	0.57
Tidal volume (mL/kg)	8 (6 - 9)	8 (7 - 9)	8 (6 - 9)	
Noradrenaline (mcg/kg/min)	0.18 (0.13 - 0.20)	0.20 (0.10 - 0.40)	0.15 (0.10 - 0.20)	0.64
HR (bpm)	98 (81 - 110)	104 (81 - 110)	93 (72 - 99)	0.99
MAP (mmHg)	77 (69 - 84)	72 (69 - 85)	77 (68 - 81)	0.26
PPV (%)	13 (6 - 16)	16 (14 - 17)	5 (3 - 8)	< 0.001
ScvO_2_ (%)	72.1 (55.3 - 79.8)	69.3 (55.3 - 80.0)	74.6 (62.1 - -78.5)	0.77
ΔCO_2_ (mmHg)	6 (4 - 7.5)	6 (3.5 -7)	4 (2 - 5.5)	0.17
Excess base	-8.6 (-15 - -0.7)	-11.6 (-15.0 - -2.3)	-8.1 (-9.3 - 0.2)	0.14

ΔPp - peak pressure variation; SAPS 3 - Simplified Acute Physiology Score 3; SaO_2_ - arterial oxygen saturation; HR - heart rate; MAP - mean arterial pressure; PPV - pulse pressure variation; ScvO_2_ - central venous oxygen saturation; ΔCO_2_ - arteriovenous gradient of carbon dioxide. The results are expressed as n (%) or median (interquartile range) or as n (percentage).

Table 2 shows the AUC and the ΔSBP and ΔMAP cut-off points at the three different levels of PEEP for discriminate patients who were potentially responsive vs. unresponsive to volume replacement. The best performance in identifying patients with PPV ≥ 12% was observed at PEEP_2_, in which a ΔSBP of -9% was identified (AUC of 0.73, 95% confidence interval (95%CI) 0.49 - 0.79; sensitivity of 0.63, 95%CI 30.8 - 89.1; and specificity of 0.80, 95%CI 44.4 - 97.5). There was no association between ΔMAP and the estimate of fluid responsiveness by PPV at any of the PEEP levels tested.

**Table 2:** Analysis of areas under the ROC curve of the hemodynamic variables for fluid-responsiveness evaluation, according to the pulse pressure respiratory variation at the three-positive end-expiratory pressure values

Variables	Threshold (%)	AUC (95%CI)	Sensitivity (95%CI)	Specificity (95%CI)	LR+	LR-	p value
ΔSBP							
PEEP_1_ level	-3	0.63 (0.45 - 0.87)	0.64 (10.9 - 69.2)	0.70 (69.2 - 100)	2.12	0.52	0.12
PEEP_2_ level	-9	0.73 (0.49 - 0.79)	0.63 (30.8 - 89.1)	0.80 (44.4 - 97.5)	3.18	0.45	0.04
PEEP_3_ level	-8	0.63 (0.40 - 0.83)	0.73 (39.0 - 94.0)	0.61 (26.2 - 87.8)	1.82	0.45	0.30
ΔMAP							
PEEP_1_ level	-8	0.63 (0.39 - 0.82)	0.36 (10.9 - 69.2)	0.60 (26.2 - 87.8)	0.91	1.06	0.32
PEEP_2_ level	-10	0.64 (0.41 - 0.84)	0.36 (10.9 - 69.2)	0.80 (44.4 - 97.5)	1.82	0.80	0.25
PEEP_3_ level	-10	0.63 (0.39 - 0.82)	0.54 (23.4 - 83.3)	0.82 (55.5 - 99.7)	5.45	0.51	0.34

AUC - area under the curve; 95%CI - 95% confidence interval; LR - likelihood ratio; ΔSBP - systolic blood pressure variation; PEEP_1_ - increase in positive end-expiratory pressure from 5 to 10cmH_2_O; ΔSBP PEEP_2_ - increase in positive end-expiratory pressure from 5 to 15cmH_2_O; PEEP_3_ - increase in positive end-expiratory pressure from 5 to 20cmH_2_O; ΔMAP - mean arterial pressure variation.

The correlation coefficient between PPV and ΔMAP was determined at PEEP_1_ (r = -0.58, 95%CI -0.80 to -0.19; p = 0.006), PEEP_2_ (r = -0.44, 95%CI -0.73 to -0.01; p = 0.04), and PEEP_3_ (r = -0.41, 95%CI -0.71 to 0.02; p = 0.06). The correlation between ΔSBP and PPV according to the different PEEP levels was PEEP_1_: r = -0.60 (95%CI -0.82 to -0.23; p = 0.004); PEEP_2_: r = -0.66 (95%CI -0.85 to -0.31; p = 0.001); and PEEP_3_: r = -0.36 (95%CI -0.68 to 0.08; p = 0.10).

**Figure 1:**
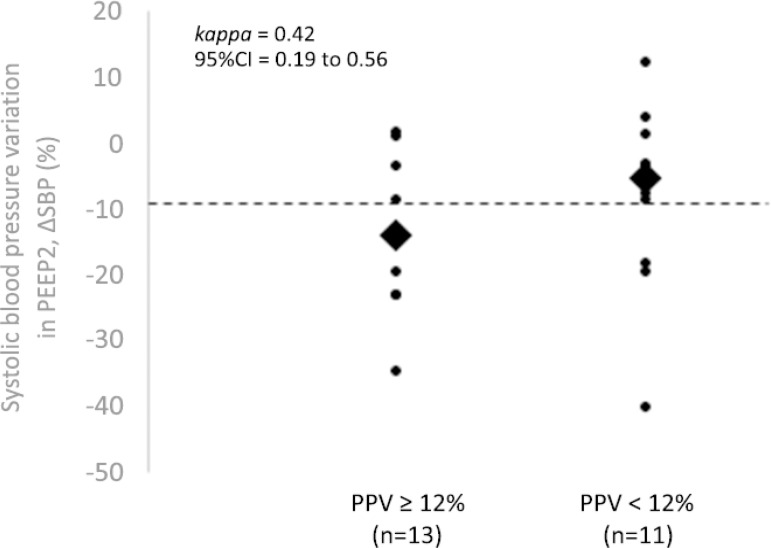
Concordance of systolic blood pressure variation (increase in positive end-expiratory pressure level 2) with pulse pressure variation ≥ 12% for identifying patients potentially responsive to volume. PEEP_2_ - increase in positive end-expiratory pressure from 5 to 15cmH_2_O; ΔSBP - variation in systolic blood pressure; 95%CI - 95% confidence interval; PPV - pulse pressure variation.

The agreement between the best-performing variable (ΔSBP) and PPV ≥ 12% for identifying patients responsive to volume was moderate (kappa = 0.42; 95%CI 0.19 to 0.56) (Figure 1).

**Figure 2:**
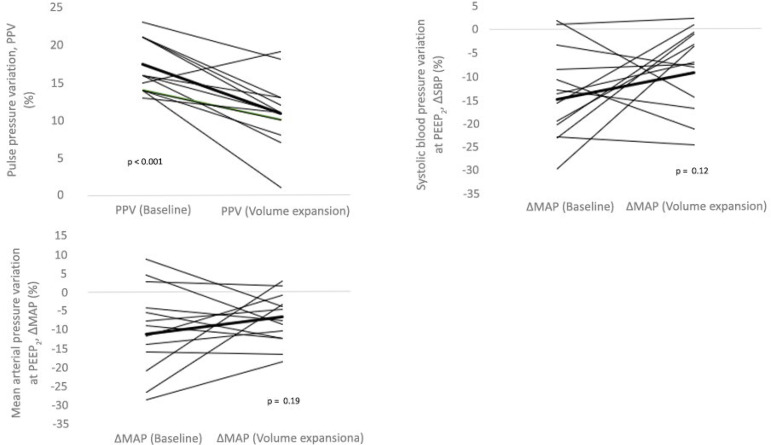
Individual responses to volume expansion in the form of pulse pressure variation, systolic blood pressure variation, and mean arterial pressure variation in 15 patients. The highlighted lines in bold show the mean values found for each parameter before and after volume expansion. The p values were obtained from the Wilcoxon test. PPV - pulse pressure variation; ΔSBP - systolic blood pressure variation; PEEP_2_ - increase in positive end-expiratory pressure from 5 to 15cmH_2_O; ΔMAP - mean arterial pressure variation.

Among the 13 patients with PPV ≥ 12%, 12 (92.3%) showed a decrease in PPV after volume expansion, while six (40.0%) had a decrease in SBP and seven (46.7%) had a decrease in MAP. The median reduction in PPV went from 16% (14% to 21%) to 11% (9% to 13%), with p < 0.001. At the same time, ΔSBP ranged from -8% (-12% to -2%) to -4% (-8% to -2%), with p = 0.26, while ΔMAP ranged from -4% (-10% to -1%) to -5% (-9% to -2%), with p = 0.34 (Figure 2).

Table 3 shows that there was a decrease in SBP and MAP during PEEP elevation at both PEEP_2_ (p = 0.006 and p = 0.009, respectively) and PEEP_3_ (p < 0.001). Seven (29.2%) patients had SBP < 90mmHg at PEEP_2_ and 10 (41.6%) had SBP < 90mmHg at PEEP_3_. Regarding MAP, five (20.8%) patients had values < 60mmHg at PEEP_2_ and seven (29.2%) had values < 60mmHg at PEEP_3_. The inspiratory pressures were higher starting at PEEP_2_, and the plateau pressure exceeded 30cmH_2_O at PEEP_3_.

**Table 3:** Ventilatory and hemodynamic variables during transient elevations of the positive end-expiratory pressure to three levels

Variables	Baseline PEEP 5cmH_2_O	PEEP_1_ Level 10cmH_2_O	PEEP_2_ level 15cmH_2_O	PEEP_3_ level 20cmH_2_O
Ventilatory				
Peak pressure in cmH_2_O	22 (19 - 24)	27 (24 - 28)*	34 (29 - 36)*	40 (37 - 45)*
Plateau pressure in cmH_2_O	15 (13 - 18)	20 (18 - 22)*	26 (24 - 28)*	33 (31 - 37)*
Hemodynamic				
HR (bpm)	98 (81 - 110)	99 (81 - 109)	99 (83 - 110)	100 (83 - 110)
CVP (mmHg)	7.5 (5 - 11.5)	10 (6 - 12)	10.5 (8 - 13.5)	12.5 (8 - 14)
SBP (mmHg)	115 (123 - 127)	113 (94 - 122)	101 (88 - 115)	93 (77 - 109)
MAP (mmHg)	77 (69 - 84)	77 (69 - 82)	73 (66 - 80)†	69 (58 - 75)†
PPV (%)	13 (6 - 16)	14 (11 - 17)	16 (11 - 24)†	19 (12 - 26)*
SBP < 90mmHg	0	2 (8.3%)	7 (29.2)†	10 (41.6)*
MAP < 60mmHg	0	1 (4.1%)	5 (20.8)†	7 (29.2)*

PEEP - positive end-expiratory pressure; PEEP_1_ - increase in positive end-expiratory pressure from 5 to 10cmH_2_O; PEEP_2_ - increase in positive end-expiratory pressure from 5 to 15cmH_2_O; PEEP_3_ - increase in positive end-expiratory pressure from 5 to 20cmH_2_O; HR - heart rate; CVP - central venous pressure; SBP - systolic blood pressure; MAP - mean arterial pressure; PPV - pulse pressure variation.

* p < 0.001;

† p < 0.01. Results expressed as mean ± standard deviation or n (%).

## DISCUSSION

The present findings do not demonstrate a relationship between PPV and decreased blood pressure (ΔSBP or ΔMAP) caused by increased PEEP as an indicator of fluid responsiveness in patients with septic shock. Thus, the effects of increased PEEP on blood pressure to guide volume replacement should be further investigated.

Pulse pressure variation is a widely known and reliable substitute marker for assessing volume responsiveness.^(11)^ The limitations of this method have motivated the search for alternatives. The increase in PEEP displaces the cardiac function curve and reduces ventricular filling, CO and, consequently, blood pressure.^(3)^ These changes are more prominent in hypovolemic patients, who tend to have a greater need for fluids and vasopressors to restore hemodynamic stability.^(3)^ Thus, it is understood that the fluid status is the basis of hemodynamic tolerance to acute PEEP increases.^(12)^ Based on this physiological foundation, changes in PPV through the application of PEEP could be used to infer changes in CO and assist in the determination of fluid responsiveness status.^(6)^ Even so, PPV has limitations that should be considered. As an alternative that would help circumvent some of these shortcomings, a proposal was made to relate the reductions in MAP and PPV through the elevation of PEEP to identify the fluid responsiveness status, and it was concluded that an 8% MAP reduction by increasing the PEEP from 10 to 20cmH_2_O could discriminate between responsive and unresponsive patients.^(4)^

The decrease in blood pressure (both MAP and SBP) in the presence of progressive PEEP application did not safely differentiate patients with PPV ≥ 12% from patients with PPV < 12%, regardless of the PEEP level adopted. Wilkman et al.^(4)^, on the contrary, found an AUROC of 0.91 (95%CI 0.77 - 1.00) for ΔMAP and 0.82 (95%CI 0.64 - 1.00) for ΔMAP, whereas the highest AUROC observed in this study was 0.73 (95%CI 0.49 - 0.79) for ΔSBP at the PEEP_2_ level, which demonstrated only a moderate ability to discriminate potentially responsive from unresponsive patients. The additional increase in PEEP to 20cmH_2_O did not increase the accuracy of the method.

The methods used by the two studies to differentiate responsive from unresponsive patients were different, which could partially explain the discrepancy in the results. Even though the variation in CO in a volume test is the gold standard, PPV reproduces this ideal technique with sensitivity and specificity greater than 90%. In turn, although a drop in blood pressure and CO is expected in response to the increase in PEEP, the blood pressure levels are subject to sympathetic compensation and may not correspond directly to the CO fluctuation. In situations of hypovolemia, for example, the decrease in CO is often hidden by normal values of blood pressure, as large contractions of blood volume are needed for low output to be reflected in reduced blood pressure.^(5)^ Thus, blood pressure may be of limited worth during hemodynamic evaluation in hypovolemic patients. This limitation is highlighted by the finding that there was no change in the ΔMAP (p = 0.18) or ΔSBP (p = 0.14) after volume expansion among the 13 patients with PPV ≥ 12%, at the same time that PPV decreased from 16% to 11% (p < 0.001). In addition, 12 of the 13 volume-responsive patients showed a reduction in PPV, while ΔMAP and ΔSBP varied in half of these responsive patients (Figure 2).

In the present study, 45.8% of patients had septic shock in the abdomen. The potential influence of intra-abdominal hypertension on diagnostic capacity should be considered.^(13)^ Recently, a study of ventilated patients with circulatory failure of all causes evaluated the relationship between intra-abdominal pressure and the end-expiratory diameter of the inferior vena cava, and it showed a significant interaction of these variables when the intra-abdominal pressure was > 12mmHg.^(13)^ Thus, high intra-abdominal pressure may be a confounding factor in predicting fluid responsiveness in ICU patients.^(13.14)^

In previous studies that addressed dynamic measures, such as PPV in septic shock, there is no evidence that the infectious site significantly influences the dynamic measurements in predicting fluid responsiveness, except in cases where there is associated intra-abdominal hypertension. In this study, eligible patients were excluded if there was suspicion or confirmation of increased intra-abdominal pressure.

Elevated PEEP may exacerbate hemodynamic effects in unstable patients, especially those with depleted intravascular volume.^(1,7,15)^ Although we did not use PEEP levels as high as those used in alveolar recruitment maneuvers,^(7)^ levels of 15 to 20cmH_2_O caused hypotension in a considerable portion of patients (Table 3), which demonstrated that increasing PEEP to quantify the fluid responsiveness status may not be safe.

This study has some limitations, including the fact that no variations in CO were assessed after infusion of fluids to check fluid responsiveness, as invasive or minimally invasive monitoring of CO was not a routine procedure in the participating hospitals. For this reason, patients were classified according to the PPV value (≥ 12% or < 12%),^(12.16)^ which may have affected the comparability with similar studies.^(4)^ Likewise, the comparison of these results with those of studies that evaluated the behavior of blood pressure after PEEP elevation may be difficult because a specific method of PEEP progression was used. Although this study dealt specifically with patients with septic shock, the number of subjects analyzed was small, which may limit the interpretation of the results, a commonality among studies on fluid-responsiveness markers.^(1,4,6,9,12,16)^

These results did not reproduce some previous findings,^(4)^ which may signal the need for further studies.

## CONCLUSION

The decrease in blood pressure in response to the increase in positive end-expiratory pressure did not reliably reflect the behavior of pulse pressure respiratory variation in the effort to identify the fluid responsiveness status, in addition to causing hypotension in a considerable portion of the patients.

## References

[1] Kang WS, Kim SH, Kim SY, Oh CS, Lee SA, Kim JS (2014). The influence of positive end-expiratory pressure on stroke volume variation in patients undergoing cardiac surgery: An observational study. J Thorac Cardiovasc Surg.

[2] Michard F, Teboul JL (2002). Predicting fluid responsiveness in ICU patients: a critical analysis of the evidence. Chest.

[3] Kim N, Shim JK, Choi HG, Kim MK, Kim JY, Kwak YL (2016). Comparison of positive end-expiratory pressure-induced increase in central venous pressure and passive leg raising to predict fluid responsiveness in patients with atrial fibrillation. Br J Anaesth.

[4] Wilkman E, Kuitunen A, Pettilä V, Varpula M (2014). Fluid responsiveness predicted by elevation of PEEP in patients with septic shock. Acta Anaesthesiol Scand.

[5] Westphal G, Garrido Adel P, de Almeida DP, Rocha e Silva M, Poli-de-Figueiredo LF (2007). Pulse pressure respiratory variation as an early marker of cardiac output fall in experimental hemorrhagic shock. Artif Organs.

[6] Michard F, Chemla D, Richard C, Wysocki M, Pinsky MR, Lecarpentier Y (1999). Clinical use of respiratory changes in arterial pulse pressure to monitor the hemodynamic effects of PEEP. Am J Respir Crit Care Med.

[7] Cavalcanti AB, Suzumura EA, Laranjeira LN, Paisani DM, Damiani LP, Guimarães HP, Writing Group for the Alveolar Recruitment for Acute Respiratory Distress Syndrome Trial (ART) Investigators (2017). Effect of lung recruitment and titrated positive end-expiratory pressure (PEEP) vs low PEEP on mortality in patients with acute respiratory distress syndrome: a randomized clinical trial. JAMA.

[8] Rhodes A, Evans LE, Alhazzani W, Levy MM, Antonelli M, Ferrer R (2017). Surviving Sepsis Campaign: International Guidelines for Management of Sepsis and Septic Shock: 2016. Intensive Care Med.

[9] Yang X, Bi Du (2014). Does pulse pressure variation predict fluid responsiveness in critically ill patients? A systematic review and meta-analysis. Crit Care.

[10] Subbe CP, Duller B, Bellomo R (2017). Effect of an automated notification system for deteriorating ward patients on clinical outcomes. Criti Care.

[11] Michard F, Chemla D, Teboul JL (2015). Applicability of pulse pressure variation: how many shades of grey?. Crit Care.

[12] Westphal GA, Silva E, Gonçalves AR, Caldeira Filho M, Poli-de-Figueiredo LF (2009). Pulse oximetry wave variation as a noninvasive tool to assess volume status in cardiac surgery. Clinics (Sao Paulo).

[13] Vieillard-Baron A, Evrard B, Repessé X, Maizel J, Jacob C (2018). Limited value of end-expiratory inferior vena cava diameter to predict fluid responsiveness impact of intra-abdominal pressure. Intensive Care Med.

[14] Jacques D, Bendjelid K, Duperret S, Colling J, Piriou V, Viale JP (2011). Pulse pressure variation and stroke volume variation during increased intra-abdominal pressure: an experimental study. Critical Care.

[15] Biasi M, Lanchon R, Sesay M, Le Gall L, Pereira B, Futier E, Nouette-Gaulain K (2017). Changes in stroke volume induced by lung recruitment maneuver predict fluid responsiveness in mechanically ventilated patients in the operating room. Anesthesiology.

[16] Westphal GA, Silva E, Caldeira Filho M, Roman Gonçalves AR, Poli-de-Figueiredo LF (2006). Variation in amplitude of central venous pressure curve induced by respiration is a useful tool to reveal fluid responsiveness in postcardiac surgery patients. Shock.

